# Active case finding for tuberculosis in tea gardens of Bangladesh: A cross-sectional survey

**DOI:** 10.1371/journal.pone.0333662

**Published:** 2025-09-29

**Authors:** Arifa Nazneen, Tanjina Rahman, Aazia Hossain, Anjan Kumar Saha, Ahammad Shafiq Sikder Adel, Asif Mujtaba Mahmud, Khalequ Zaman, Azharul Islam Khan, Aung Kya Jai Maug, Shahriar Ahmed, Sayera Banu

**Affiliations:** 1 International Centre for Diarrhoeal Disease Research, Bangladesh (icddr, b), Dhaka, Bangladesh; 2 United Hospital Limited, Dhaka, Bangladesh; The University of Georgia, UNITED STATES OF AMERICA

## Abstract

Tea garden populations with poor socio-economic status are at risk of developing TB. The active case finding (ACF) approach is effective in finding TB among the people at risk. We have conducted ACF for TB to find people with presumptive TB in the tea gardens of Sylhet division to identify TB disease. It was a cross-sectional survey conducted at the household level, in the randomly selected three tea gardens of Sylhet division. The selected population was 20,215 and was screened for TB presumptive symptoms between July and Oct 2022. Each presumptive had required TB testing, which includes GeneXpert MTB/RIF (Xpert) or Sputum microscopy, X-ray, and Fine Needle Aspiration Cytology (FNAC) if there was gland swelling. Around 99.6% (20,127/20,215) of the surveyed population were screened for TB. Among the screened population, gender distribution was almost equal, and 34% had no education. And people with presumptive TB were 0.8%; among them, most (91%) were pulmonary TB presumptive with male predominance. Among all presumptive, 87.4% had a cough for ≥ 14 days and 78% had a fever. We identified a total of 17 pulmonary TB; among them, bacteriologically confirmed TB were only 6 (35.3%). The estimated proportion of TB among the surveyed population was 0.10%. The findings suggest a need for sustained TB screening activities integrated with community involvement.

## Introduction

Early diagnosis of tuberculosis (TB) and successful treatment prevent millions of new TB infections and deaths each year, though there are still persistent gaps in TB detection and treatment [1]. According to the World Health Organization’s (WHO) Global TB Report 2024, 303,686 people with TB (PwTB) were notified and registered with the National Tuberculosis Programme (NTP) in Bangladesh, with an estimated 20% missing TB in 2023 [2]. Gaps between the estimated and reported number of new TB are due to underdiagnosis and underreporting of detected TB [3]. Moreover, even after diagnosis, PwTBs often experience prolonged delays before initiation of correct treatment, which further exacerbates disease transmission [4,5]. Expansion and strengthening of TB care services to address this gap is much needed. Active Case Finding (ACF) is a WHO-recommended systematic screening process where communities or populations at risk of TB are actively searched for TB symptoms, individuals identified with presumptive TB are tested and clinical assessments are done for TB [6,7].

Ending the TB epidemic requires a multi-sectoral response, including engagement of civil society, communities, diverse government sectors, and the private sector [8]. TB key populations, such as those with poverty, facing social marginalization, and living in remote areas, often face socio-cultural barriers limiting their access to health services, and targeted interventions are crucial to reach these groups and ensure access to TB care [9].

ACF among these key populations can improve their access to healthcare and achieve improved health outcomes [10]. Areas with high TB incidence can serve as TB infection reservoirs and facilitate transmission inside the larger community [10,11]. Therefore, targeting TB hotspot areas can be an effective way of reducing TB incidence locally rather than interventions among mass population [12]. Mathematical models have also suggested that ACF could increase TB detection [6,13]. A study in Vietnam revealed that community-based ACF, through engagement of Community Health Workers with access to chest X-ray screening and GeneXpert MTB/RIF (Xpert) testing, can effectively identify community people with TB at high-burden settings [14].

Since TB is still a public health concern among marginalized groups, such as in ethnic communities, tea garden populations, and hard-to-reach areas where poor living and working conditions contribute to heightened vulnerability [15]. Tea garden workers with their families often live in overcrowded and poorly ventilated housing with limited access to healthcare, and experience poor nutrition, leading to the risk of TB infection and active disease [15–17]. Study conducted in the tea gardens of Assam in India reported higher TB prevalence than national averages [18]. Moreover, stigma, poor health literacy, delay in diagnosis, and treatment initiation exacerbate disease transmission in the tea garden [17].

In Bangladesh, tea garden workers face socio-economic disadvantages and are often overlooked, resulting in limited access to healthcare services. Their poor socio-economic condition and deprived livelihoods lead to social exclusion [21]. Their poor living make them TB risk populations [19]. Previous studies in a systematic reviews consistently found that there was higher tendency of developing TB among lower income group [20].

In collaboration with NTP, USAID’s Alliance for Combating TB in Bangladesh (ACTB) Activity aimed to find TB in the targeted (high-risk) community through the ACF approach. Therefore, we conducted a survey in tea gardens of the Sylhet division of Bangladesh and implemented ACF to detect new TB among the surveyed population.

## Materials and methods

**Fig 1:**
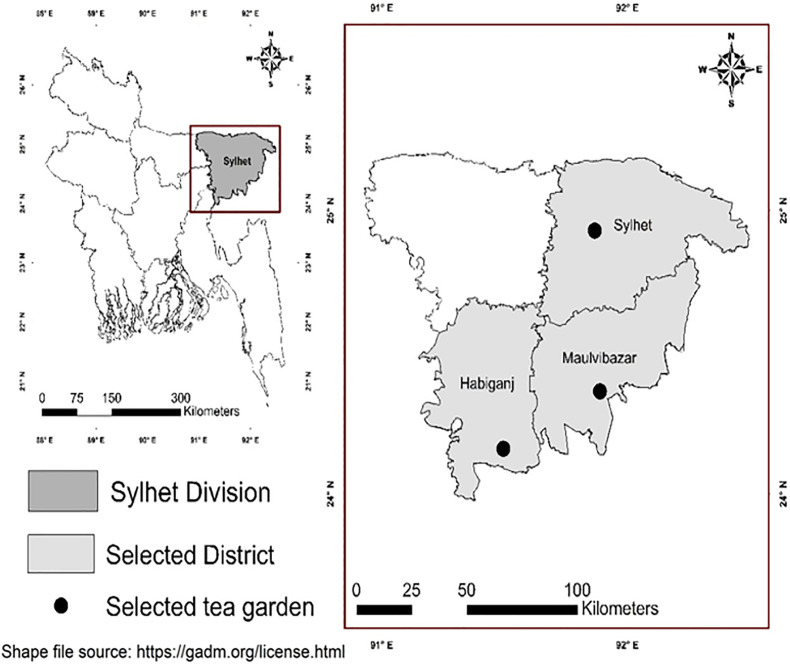
Map of Bangladesh showing three districts of Sylhet division where the selected tea gardens are located. [Reprinted from GADM (https://gadm.org/license.html) under a CC BY license, with permission from Robert Hijmans, original copyright ©GADM].

The study design was cross-sectional and was conducted through a community survey to identify people with presumptive TB through ACF in the tea gardens under the Sylhet division of Bangladesh. We collected survey data and baseline information on the population through a structured questionnaire and conducted TB screening using NTP-approved screening tool or criteria (Table 1) at the tea garden households (HH) from July 20, 2022, to October 30, 2022. Tea garden employers provide housing for workers in the form of labor lines or small houses with limited facilities and poor access to education, health, water and sanitation [21]. Sylhet division has 184 tea gardens in three out of its four districts. Hence, we collected a list of tea gardens under each district and randomly picked one garden from each district. Therefore, three tea gardens were selected, one each from Sylhet, Moulvibazar, and Habiganj districts (Fig 1). We marked the selected tea gardens as tea garden-1/TG-1 (Sylhet), tea garden-2/TG-2 (Moulvibazar), and tea garden-3/TG-3 (Habiganj). A comprehensive list of all tea gardens (n = 184) in the Sylhet division was first compiled (listing frame) using data obtained from office-registered records. Among them, we recorded 19 from the Sylhet district, 136 tea gardens from the Moulvibazar district, and 29 from the Habiganj district. From these lists, gardens were grouped according to their respective districts. A simple random sampling method was applied to select one garden within each district. We assigned a random number to each garden, and then documented and recorded the chosen garden in each district before initiating any field activities. The randomization was conducted using a computer-generated random number sequence in Microsoft Excel (RAND function), and the process was followed by the study statistician, who was involved in both sampling process and field operations. During data collection, she frequently visited the study data collection sites for monitoring and supervision. She ensured that the random selection process was followed correctly to avoid any biases and deviations from the study method.This approach was employed to ensure representativeness while maintaining logistical feasibility for fieldwork in geographically distinct districts of the Sylhet division.

**Table 1:** TB screening criteria for adults and children.

Age group	Sl.	TB screening indicators	Criteria to be TB presumptive
Adult (≥15 Yrs.)	**1**	Persistent cough for ≥2 weeks	• Indicator 1 with or without any other (2–6) • Indicator 5 with or without any other (1–4; 6) • Indicator 6 with any other (1–5) • Indicator 2 + 3 + 4 together with or without any other
	**2**	Fever for ≥2 weeks	
	**3**	Night sweats	
	**4**	Weight loss	
	**5**	Lump in the neck, armpit, or groin	
	**6**	History of contact with TB patient in last 12 months	
Child (0–14 Yrs.)	**1**	Persistent cough for ≥ 2 weeks,	• If there were any children with any one of the six indicators, they were identified as presumptive TB
	**2**	Fever for ≥ 2 weeks	
	**3**	Weight loss/ failure to gain weight	
	**4**	Reduced playfulness/ fatigue	
	**5**	Lump in the neck, armpit, or groin	
	**6**	History of contact with TB patient in last 12 months	

The positivity of TB was reported as 12% among tested presumptive under the public-private mix initiative of icddr,b in 2019 [22]. Recent research conducted in India on indigenous populations found that 3.4% were presumptive for TB among the screened population [23]. However, based on early internal analyses of ongoing program data (ACTB-ACF activities) implemented in six out of eight divisions across Bangladesh in primary, secondary, and tertiary health facilities (not yet published)and with the practical experience of national TB program experts familiar with similar rural and hard-to-reach populations, we assumed to get around 2% of people with presumptive TB among the screened population of the tea gardens with around 5% TB detection rate.

We conducted face-to-face interviews (S1 File) with the participants. Our respondents at HHs were the HH head or his/her representative, an adult (age > 18 years) family member. We also performed systematic screening for TB among all listed populations from each HH, with the consent of the respective HH respondents, using a structured screening tool or criteria (S2 File) approved by NTP. We asked for cough (≥14 days), fever (≥14 days), weight loss, night sweats (adult), less playfulness (child), gland swelling, and TB contacts to identify people with presumptive TB. The following table (Table 1) contains the indicators and criteria for identifying the TB presumptive according to age.

We also collected socio-demographic data (age, sex, education, occupation, income, sanitation, and TB-related information) from the respondents. Data was collected digitally using tablets. Each health worker screened around 60 people daily. When any presumptive TB was found, they were counselled for TB testing. Our trained field team provided them sputum collection cup and instructed them to collect a sputum sample in the morning. The following day, the field staff collected the sputum cup with the sample from the presumptive individual. The sample was transported to the TB testing lab or government healthcare facility adjacent or nearest to the garden for testing (sputum Xpert or Sputum microscopy, X-ray, Fine Needle Aspiration Cytology (FNAC) if there was gland swelling). The team members maintained standard biosafety measures while interviewing participants and transporting samples.

We also arranged X-rays for the presumptive by providing transport facilities. All X-rays were evaluated by an expert panel consisting of three radiologists through an online teleradiology platform. Extra-pulmonary TB presumptive were referred to the nearest government healthcare facility for further evaluation and testing.

**Fig 2:**
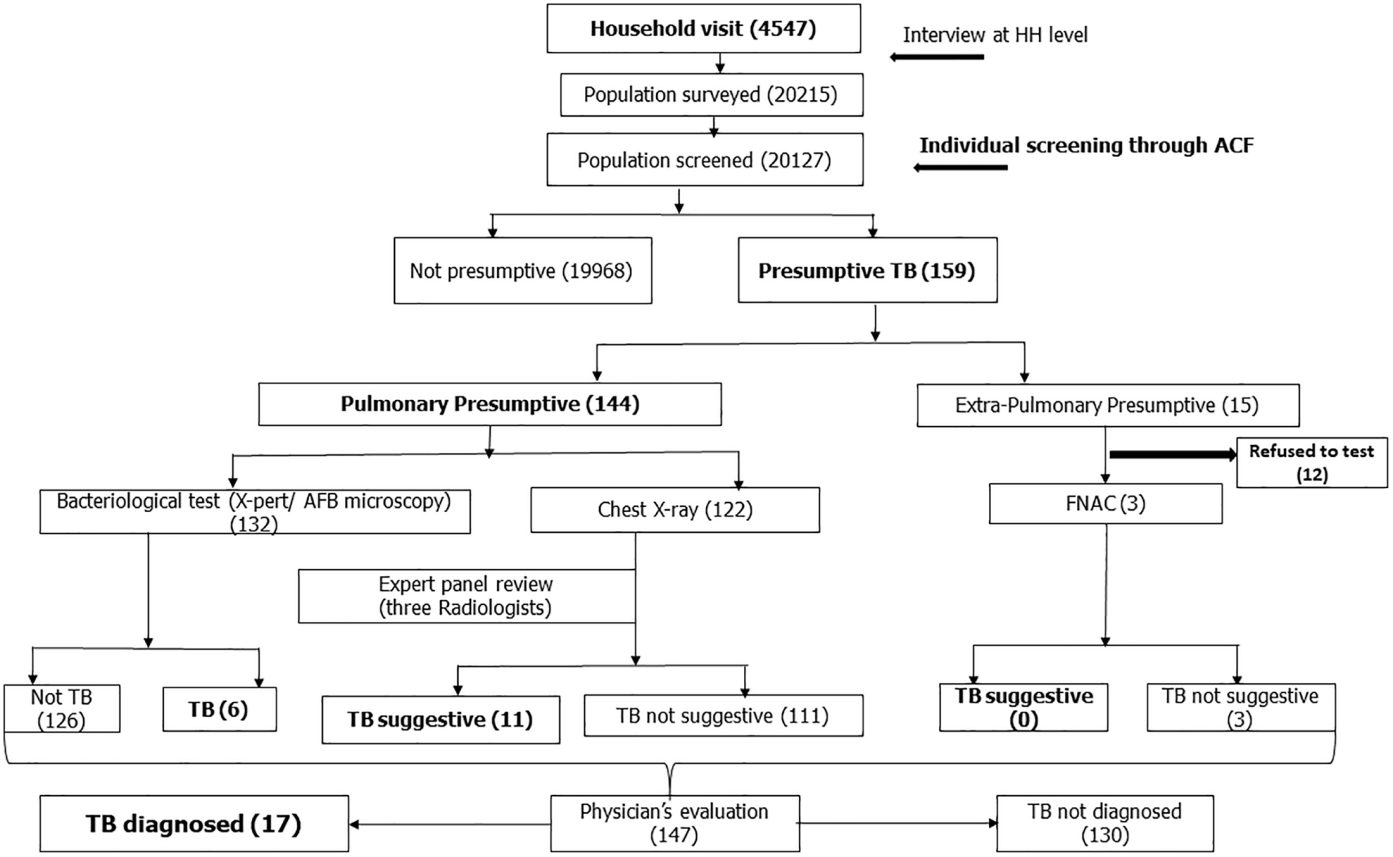
Flow chart of study method.

Participants were informed immediately after collecting test results and were accompanied to the physician with the reports. When physicians diagnosed an individual with TB based on reports and clinical findings, the team ensured prompt TB treatment initiation from the corresponding Directly Observed Treatment providers. Finally, the clinically diagnosed (CD) pulmonary TB were those whose X-rays were suggestive of TB (when at least two out of three radiologists reported TB in the X-ray report) by the expert panel and were diagnosed as TB by the attending physician. The whole method of the survey is shown in a flow chart (Fig 2).

### Data analysis

We analyzed the data using the statistical software Stata/SE 17. Descriptive statistics, such as frequency, proportions, mean and median, were used to report the socio-demographic details, symptom profile, and TB data. We calculated the wealth index by using the principal component analysis method [24]. It is a composite measure of a household’s cumulative living standard [25]. Each household was assigned a standardized score for each asset and the collective score differs from one another depending on whether the household owned the assets or not. We considered the assets, e.g., monthly HH income, expenditure, number of rooms in HH, type of toilet facility used, toilet shared or not, main source of drinking water, HH owned electricity, television, mobile phone, refrigerator, fan, computer/laptop etc. Generally, a variable with a positive score is associated with higher socio-economic status (SES), and conversely, a variable with a negative factor score is associated with lower SES. The resulting asset scores are standardized concerning a standard normal distribution with a mean of 0 and a standard deviation of 1. The sample is then divided into four population quintiles with the same number of individuals in each. These standardized scores are then used to create the breakpoints that defined wealth quintiles as: poorest, poor, middle and rich. Proportion of TB detection was calculated among people with presumptive TB. The population-based detection rate was obtained by extrapolating the rates in 100,000 population. We performed χ2 test for categorical data and an independent sample t-test for continuous data to identify group differences. Statistical significance was determined at p-value < 0.05.

### Ethical consideration

This study was approved by the Institutional Review Board of icddr,b’s Research Review Committee (RRC) and Ethical Review Committee (ERC), the IRB number was PR#22051. Informed written voluntary consent was obtained from all household heads. Participant’s confidentiality and anonymity were maintained.

## Results

We enrolled almost 99.9% (4,547/4,553) of the total HHs under the survey area (Table 2). The total population we surveyed was 20,215 (Table 3). The average HH size was 4.4 (median: 4; IQR: 3–5).

**Table 2:** Socio-demographic information of the surveyed households and participants.

Characteristics		TG 1, *n = 848 (%)	TG 2, *n = 1469 (%)	TG 3, *n = 2230 (%)	Total, *n = 4547 (%)	P-value^¶^
Monthly income of HH	500-5000	151 (17.8)	557 (37.9)	55 (26.7)	1303 (28.7)	<0.01
	5001-10000	475 (56)	655 (44.6)	1103 (49.5)	2233 (49.1)	
	10001-20000	191 (22.5)	175 (11.9)	409 (18.3)	775 (17)	
	20001-50000	21 (2.5)	61 (4.2)	72 (3.2)	154 (3.4)	
	>50000	1 (0.1)	3 (0.2)	3 (0.1)	7 (0.2)	
	Missing	9 (1.1)	18 (1.2)	48 (2.2)	75 (1.7)	
Median no. of family members per HH (IQR)		4 (3, 5)	4 (4, 6)	4 (3, 5)	4 (3, 5)	
Wealth quintiles	Poorest	251 (29.6)	272 (18.5)	730 (32.7)	1253 (27.6)	<0.01
	Poor	172 (20.3)	360 (24.5)	499 (22.4)	1031 (22.7)	
	Middle	180 (21.2)	383 (26.1)	573 (25.7)	1136 (25.0)	
	Rich	245 (28.9)	454 (30.9)	428 (19.2)	1127 (24.8)	
Mobile phone access (Yes)		709 (83.6)	1327 (90.3)	1779 (79.8)	3815 (84.0)	<0.01
Toilet facility used	Improved Pit latrine	567 (66.9)	863 (58.7)	1148 (51.5)	2578 (56.7)	<0.01
	Open Pit latrine	98 (11.6)	320 (21.8)	373 (16.7)	791 (17.4)	
	Toilet without a septic tank	63 (7.4)	132 (9)	91 (4.1)	286 (6.3)	
	Hanging toilet	17 (2)	23 (1.6)	51 (2.3)	91 (2)	
	Flushed toilet with septic tank	13 (1.5)	20 (1.4)	13 (0.6)	46 (1)	
	Other	1 (0.1)	2 (0.1)	8 (0.4)	11 (0.2)	
	No toilet facility	80 (9.4)	92 (6.3)	501 (22.5)	673 (14.8)	
	Missing	9 (1.1)	17 (1.2)	45 (2)	71 (1.6)	

*n = Total number of households interviewed, TG = Tea Garden, HH = Household, % = Column percentage

^¶^P-values indicate group comparisons using Chi-square or Fisher’s exact test

**Table 3:** Socio-demographic information of the surveyed participants.

Characteristics		TG 1, n = 3744 (%)	TG 2, n = 6837 (%)	TG 3, n = 9634 (%)	Total, n = 20215 (%)	P-value^¶^
Age in years	<5	317 (8.5)	516 (7.5)	889 (9.2)	1722 (8.5)	<0.01
	5-14	676 (18.1)	1108 (16.2)	1731 (18)	3515 (17.4)	
	15-24	945 (25.2)	1540 (22.5)	1866 (19.4)	4351 (21.5)	
	25-44	1099 (29.4)	1975 (28.9)	2904 (30.1)	5978 (29.6)	
	45-64	536 (14.3)	1280 (18.7)	1637 (17)	3453 (17.1)	
	65+	171 (4.6)	418 (6.1)	607 (6.3)	1196 (5.9)	
Gender	Male	1834 (49)	3431 (50.2)	4836 (50.2)	10101 (49.97)	0.412
	Female	1910 (51.0)	3406 (49.8)	4798 (49.8)	10114 (50.03)	
Education	No education	1349 (36)	2091 (30.6)	3489 (36.2)	6929 (34.3)	<0.01
	Up to Primary	1341 (35.8)	2224 (32.5)	3578 (37.1)	7143 (35.3)	
	Up to SSC	911 (24.3)	1976 (28.9)	2143 (22.2)	5030 (24.9)	
	HSC	126 (3.4)	426 (6.2)	338 (3.5)	890 (4.4)	
	Graduation^+^	17 (0.5)	120 (1.8)	86 (0.9)	223 (1.1)	
Occupation	Tea garden worker	1604 (42.8)	1873 (27.4)	1970 (20.4)	5447 (27.0)	<0.01
	Unemployed	399 (10.7)	1114 (16.3)	1361 (14.1)	2874 (14.2)	
	Student	889 (23.7)	1576 (23.1)	2057 (21.4)	4522 (22.4)	
	Homemaker	288 (7.7)	906 (13.3)	1028 (10.7)	2222 (11.0)	
	Others	564 (15.1)	1368 (20)	3218 (33.4)	5150 (25.5)	
History of TB (Yes)		69 (1.8)	79 (1.2)	264 (2.7)	412 (2.0)	<0.01
BCG vaccine (Yes)		3566 (95.2)	6231 (91.1)	8731 (90.6)	18528 (91.7)	<0.01
Smoking history (Yes)		324 (8.7)	279 (4.1)	1115 (11.6)	1718 (8.5)	<0.01
Diabetes (Yes)		19 (0.5)	27 (0.4)	22 (0.2)	68 (0.3)	<0.01

*n = Total number of populations screened, TG = Tea Garden, % = Column percentage

^¶^P-values indicate group comparisons using Chi-square or Fisher’s exact test

Most of the HH population (29.6%) was between the ages of 25 and 44. Gender distribution was almost equal (Table 3). Most of the participants (92%) were from the Hindu community, and 8% were Muslims. About 78% of the HHs had a monthly income of Taka 500–10,000, whereas half of the HHs (49.2%) had a monthly income of Taka 5,001–10,000. About 84% of the HHs had access to mobile phones. Majority of HHs (90%) used tube-wells as the source of drinking water. Around 57% of the HHs had improved toilet facilities. Around 50% of HHs belonged to the poor and poorest wealth quintile (Table 2).

About 34% of HH members had no education, and 35% had completed primary education. Around 27% of HH members were tea garden workers (Table 3). Most of the HH members (92%) had received BCG vaccinations. Only 2% of the surveyed population had a history of TB. Among the HH members, 10% reported being alcohol drinkers, 18% reported a habit of substance use, and 8.5% reported being tobacco smokers. Among all participants, only 0.3% had reported having diabetes.

We screened 99.6% (20,127/20,215) of individuals for TB and 0.8% (159/20,127) were presumptive TB. Among the presumptive, most (91%, 144/159) were pulmonary TB presumptive (Table 4), with male predominance (62%). Most of the presumptive (30%) were between 45 and 65 years of age. Table 4 contains clinical information on the presumptive TB, and Table 5 shows the information on the investigations. Most of the presumptives had a cough for ≥ 14 days (87%), fever (78%), weight loss (80%), and night sweats (54%).

**Table 4:** Age, sex, and clinical history of presumptive TB.

Characteristics		TG 1, *n = 61 (%)	TG 2, *n = 19 (%)	TG 3, *n = 79 (%)	Total, *n = 159 (%)	P-value^¶^
Presumptive (%)		61 (1.8)	19 (0.3)	79 (0.8)	159 (0.8)	
PTB Presumptive (%)		48 (78.6)	19 (100)	77 (97.5)	144 (91)	
EPTB presumptive (presence of lump) (%)		13 (21.4)	0 (0)	2 (2.5)	15 (9)	
Age in years	<5	2 (3.3%)	0 (0%)	1 (1.3%)	3 (1.9%)	0.245
	5-14	8 (13.1%)	1 (5.3%)	5 (6.3%)	14 (8.8%)	
	15-24	10 (16.4%)	3 (15.8%)	8 (10.1%)	21 (13.2%)	
	25-44	13 (21.3%)	3 (15.8%)	25 (31.6%)	41 (25.8%)	
	45-64	16 (26.2%)	8 (42.1%)	33 (41.8%)	57 (35.8%)	
	65+	12 (19.7%)	4 (21.1%)	7 (8.9%)	23 (14.5%)	
Gender	Male	38 (62.3%)	12 (63.2%)	53 (67.1%)	103 (64.8%)	0.830
	Female	23 (37.7)	7 (36.8)	26 (32.9)	56 (35.2)	
Clinical information	Cough	47 (77)	18 (94.7)	74 (93.7)	139 (87.4)	0.008
	Cough duration (Mean±SD)	21.1 ± 8.1	44.3 ± 28.1	30.6 ± 26.7	29.1 ± 23.4	
	Fever	41 (67.2)	13 (68.4)	70 (88.6)	124 (78)	0.006
	Fever duration (Mean±SD)	17.2 ± 5.5	34.6 ± 27.7	26.5 ± 24.8	24.3 ± 21.4	
	Night sweats	28 (45.9)	8 (42.1)	49 (62)	85 (53.5)	0.080
	Weight loss	44 (72.1)	14 (73.7)	69 (87.3)	127 (79.9)	0.065
	TB contact history	4 (6.6)	1 (5.3)	17 (21.5)	22 (13.8)	0.020

*n = Total number of presumptive,TG = Tea Garden, PTB = Pulmonary TB, EPTB = Extra Pulmonary TB, SD = StandardDeviation, % = Column percentage, duration expressed in days.

^¶^ P-values indicate group comparisons using Chi-square or Fisher’s exact test

**Table 5:** Investigation and results of pulmonary TB (PTB) presumptive.

Diagnostics	TG 1, *n = 48 (%)	TG 2, *n = 19 (%)	TG 3, *n = 77 (%)	Total, *n = 144 (%)	P-value^¶^
Xpert done (%)	43 (90)	14 (74)	68 (88)	125 (87)	
Xpert positive (%)	2 (5)	0 (0)	4 (6)	6 (5)	0.551
AFB microscopy done (%)	0 (0)	5 (25)	2 (3)	7 (5)	
AFB positive (%)	0 (0)	0 (0)	0 (0)	0 (0)	
X-ray done (%)	47 (99)	12 (63)	63 (82)	122 (85)	
X-ray suggestive of TB (two of the panel members suggested TB)	2	1	14	17	
Pulmonary CDTB [X-ray report + Physician’s opinion] (%)	2 (4)	1 (8)	8 (10)	11 (8)	0.624
B + , TB (%)	2 (4)	0 (0)	4 (5)	6 (4)	
Total TB (%)	4 (8)	1 (5)	12 (16)	17 (12)	

*n = Total number of pulmonary TB presumptive, PTB = Pulmonary TB, TG = Tea Garden, HH = Household, Xpert = Gene Xpert, AFB = Acid-Fast Bacillus, CD = Clinically diagnosed, B+ = Bacteriologically confirmed, % = Column percentage

^¶^ P-values indicate group comparisons using Chi-square or Fisher’s exact test

Among the identified PwTB, all had history of cough for ≥ 14 days. Moreover, almost all of them had fever accompanying their coughs (Table 6). We found six bacteriologically confirmed (B+) pulmonary TB (2 in TG-1, 4 in TG-3) positive by Xpert. Identified B + TB was 4.1% (6/144) of the total pulmonary TB presumptive. Based on the X-ray findings (through an expert panel) and local physicians’ opinion, reported pulmonary CD TB were 11 (2 in TG-1, 1 in TG-2, and 8 in TG-3). No EPTB was identified during the survey period. The total number of diagnosed TB was 17 (10.7%, 17/159), with a predominance of CD TB (64.7%, 11/17) among the identified presumptive TB. The estimated proportion of TB among the surveyed population was 100 per 100,000 during the survey.

**Table 6:** Information on people identified with TB.

Characteristics		TG 1, *n = 4 (%)	TG 2, *n = 1 (%)	TG 3, *n = 12 (%)	Total, *n = 17 (%)
Age in years	<5	0	0	0	0
	5-14	0	0	1 (8.3)	1 (5.9)
	15-24	3 (75.0)	0	0	3 (17.7)
	25-44	0	0	6 (50.0)	6 (35.3)
	45-64	1 (25.0)	0	3 (25.0)	4 (23.5)
	65+	0	1 (100)	2 (16.7)	3 (17.7)
Male		1 (25.0)	1 (100)	6 (50.0)	8 (47.1)
Clinical information	Cough	4 (100)	1 (100)	12 (100)	17 (100)
	Cough duration (Mean±SD)	23.0 ± 2.3	26.0	27.1 ± 20.8	26.1 ± 17.3
	Fever	4 (100)	1 (100)	12 (100)	17 (100)
	Fever duration (Mean±SD)	20.2 ± 7.1	14.0	27.2 ± 23.8	24.8 ± 20.4
	Night sweats	1 (25.0)	1 (100)	7 (58.3)	9 (52.9)
	Weight loss	4 (100)	0	11 (91.7)	15 (88.2)
	TB contact history	0	0	1 (8.3)	1 (5.9)

*n = Total number of TB, TG = Tea Garden, HH = Household, % = Column percentage

## Discussion

Tea garden workers are at risk of developing TB due to poor living conditions, including poverty, low education levels, crowded housing, and nutritional deficiencies, which increase their vulnerability to TB [17,18]. This study provides information on the demographic and socio-economic characteristics of tea garden communities in Bangladesh, as well as the proportion of TB in these communities. The socio-economic status of tea garden workers in Bangladesh, highlighted in an article published in 2022, aligns with the study findings [26]. The average household size and age distribution among participants correspond with national data [27,28], and BCG vaccination coverage was similar to the national level [29].

There was a lower presumptive TB identification than expected, but a similar finding was reported in a study conducted among tea garden populations in India [30]. The fewer individuals with presumptive TB could be due to door-to-door screening at a single point in time. The study found an almost equal male-to-female ratio in the surveyed population. However, among presumptive and confirmed TB, males were predominant which coincide with other studies [31–34]. Presumptive TB and confirmed TB were predominant in the higher age group, which was consistent with data from a systematic review and meta-analysis [31]. All confirmed TB and most of the presumptive TB had cough ≥ 14 days, which was the key presenting symptom for TB, consistent with the findings of an Ethiopian study [35].

There is an instruction in the national guideline for clinically diagnosed TB for children, but no definitive criteria for adults. The study found that clinically diagnosed TB was 65%, which does not match with national data (25%) or other country data [2,36,37]. And bacteriologically confirmed TB accounted for 4% of the total presumptive, but the national reports show positivity around 10% [22]. However, within a small community, this type of finding is observable (unpublished data from the community ACF of ACTB).

The estimated TB detection rate does not accurately reflect the prevalence of TB in tea gardens. Instead, it represents the proportion of TB detections achieved through the ACF approach in a cross-section of tea gardens within the Sylhet division over a specific period. The overall TB notification reported nationally in the Sylhet division was higher [37]. Available evidence shows that ACF is a feasible strategy for the detection of tuberculosis [38]. There is evidence that community approaches to TB prevention adopted by Bangladesh attained success in TB detection and better treatment outcomes [39]. Also, studies done in India found that ACF activity yields early detection of TB and increases the treatment success rate [40,41].

### Limitations

The study was conducted at a specific point of time, where a few presumptive were identified. We only tested the symptomatic individuals; therefore, we may have missed the asymptomatic or subclinical TB. Although the X-ray images were reviewed remotely by a panel, the patients were not evaluated by a pulmonologist in person. We depended on the opinion of the physicians working in local public healthcare facilities for TB diagnosis. It was a limitation not to use X-ray during screening for all participants; instead, X-ray was only performed on individuals with presumptive TB. Screening with chest X-rays could yield the detection of more TB at an early stage.

## Conclusions

The usefulness of active TB screening was emphasized in this study to identify TB among the tea garden population. The ACF approach in vulnerable communities prone to TB can bridge diagnostic gaps to achieve better health outcomes. The ACF approach detected missing people with TB at an early stage in the tea gardens. Since ACF is an effective tool for TB detection, ongoing intervention must be maintained. The findings support the scalable strategies of ACF with diagnostic supports. Strong and sustained TB surveillance in tea gardens may contribute to national efforts to end TB.

## Supporting information

S1 File Questionnaire-Baseline data collection form (Household information). (PDF)

S2 File Screening tool-Active case finding (ACF) for Adult and Child. (PDF)
