# Supplementary material for: Active case finding for tuberculosis in tea gardens of Bangladesh: A cross-sectional survey
Source: PLoS One. 2025 Sep 29;20(9):e0333662. doi: 10.1371/journal.pone.0333662 (PMC12478949; doi:10.1371/journal.pone.0333662)
Supplement: S1 File — (PDF) [file pone.0333662.s001.pdf]

Baseline data collection form (Household information)

Title: Active case finding of Tuberculosis at the Tea gardens of Sylhet division: A cross-sectional Survey

ID: (আই. ডি.) ☐☐ ☐☐☐☐ Date of interview: (স্বাক্ষাতকার গ্রহণের তারিখ)

District: (জেলা) Upazila/Thana: (উপজেলা/থানা)

Name of the Tea Garden: (চা বাগানের নাম) Lane/line number: (লেন/লাইন নম্বর)

House No: (বাড়ি নম্বর)

Name of interviewer: (সাক্ষাৎকার গ্রহণকারীর নাম) ....

Mobile no. of interviewer: (সাক্ষাৎকার গ্রহণকারীর মোবাইল) ....

1. Primary information (প্রাথমিক তথ্য)

Q no (প্রশ্ন নং)	Questions (প্রশ্ন)	Answer Options (উত্তরের বিকল্প)	Code (কোড)
1.1	Name of the respondent (উত্তরদাতার নাম)		
1.2	Age of Respondent (must be >18 yrs old) উত্তরদাতার বয়স (অবশ্যই ১৮ বছরের বেশি হতে হবে)	In years (বছরে)	
1.3	Education of the Respondent (উত্তরদাতার শিক্ষা)	Completed years of Education: No education-0, Class one-SSC:1-10, HSC-12, Graduation-14, Masters-17<br>শিক্ষার শেষ বছর: অশিক্ষিত-০, প্রথম শ্রেণী থেকে এসএসসি: ১-১০, এইচএসসি-১২, স্নাতক-১৪, স্নাতোকত্তর-১৭	
1.4	What is the relation of the household head to the respondent?<br>উত্তরদাতার সঙ্গে খানা প্রধানের সম্পর্ক কি?		
1.5	Did the household agree to participate in the survey?<br>খানা প্রধান/সদস্য কি এই গবেষণায় অংশগ্রহণ করতে ইচ্ছুক?	1= Yes (হ্যাঁ), 0 =No (না) If no: (end interview) যদি না হয়: স্বাক্ষাতকার শেষ করুন	
1.6	Name of the household (HH) head<br>খানা প্রধানের নাম		
1.7	Religion<br>ধর্ম	Muslim-1 Hindu-2, Buddist-3, Christian-4, Trival-5<br>মুসলিম-১, হিন্দু-২, বোদ্ধ-৩, খ্রিষ্টান-৪, উপজাতী-৫	
1.8	Contact No.<br>মোবাইল নম্বরঃ	☐☐☐☐☐☐☐☐☐☐☐<br>☐☐☐☐☐☐☐☐☐☐☐	

Code, Relation: 01 = head, 02 = wife or husband, 03 = son or daughter, 04 = son-in-law or daughter-in-law, 05 = grandchild, 06 = parent, 07 = parent-in-law, 08 = brother or sister, 09 = other relative, 10 = adopted/foster/stepchild, 11 = not related, 88 = don't know

কোড, সম্পর্ক: ০১=প্রধান, ০২=স্ত্রী বা স্বামী, ০৩=ছেলে/মেয়ে, ০৪=শ্যালক/শালিকা, ০৫=নাতি/নাতনী, ০৬=বাবা/মা, ০৭=শশুর/শাশুড়ী, ০৮=ভাই/বোন, ০৯=অন্যান্য আত্নীয়, ১০=দত্তক/পালিত/সৎসন্তান, ১১=সম্পর্কিত নয়, ৮৮=জানিনা

2a. Information of all members of the household (খানার সকল সদস্যদের তথ্য) [for the Ans. don't know use code-88 and for answer not applicable use 99, in case of ans. others: please specify] উত্তরের জন্য জানিনা-৮৮ কোড, প্রযোজ্য হয়-৯৯ কোড ব্যবহার করুন এবং উত্তরের ক্ষেত্রে অন্যান্য হলে, দয়া করে উল্লেখ করুন।

Sl.	2a.1 Name (নাম)	2a.2 ID (আইডি)	2a.3 Age (বয়স)	2a.4 Sex (লিঙ্গ)	2a.5 Education (শিক্ষা)	2a.6 Occupation (পেশা)	2a.7 BCG Vaccine (বিসিজি টিকা)	2a.8 Nutrition (Wt./Ht.) পুষ্টি (ওজন/উচ্চতা)	2a.9 Remarks (মন্তব্য)
	Full name (পূর্ণ নাম)	HHID+ Member no. (খানার আইড+সদস্য নং)	-Y-M-D (ব-মা-দি)	1=M/2=F/3 =3rd (১=পুঃ/২=মঃ/ ৩=তৃতীয়)	Comp. years (পূর্ণ বছর)	Write (লিখুন)	1= Yes (হ্যাঁ) 0 =No (না)	1-Aveg. ,2-Over,3-Under (১-মাঝারি, ২-বেশি, ৩-কম)	Any added information (কোন সংযুক্ত তথ্য)

Occupation:1=Tea Garden worker, 2-Unemployed, 3-Student, 4-Child, 5-house wife 77=Others (specify) পেশাঃ ১=চা বাগানের শ্রমিক, ২=বেকার, ৩=ছাত্র, ৪=শিশু, ৫=গৃহিনী, ৭৭=অন্যান্য (ব্যাখ্যা করুন)

2b. Information of all members of the household (খানার সকল সদস্যদের তথ্য) [for the Ans. don't know use code-88 and for answer not applicable use 99, in case of ans. others: please specify] এই উত্তরের জন্য কোড ৮৮ জানিনা এবং কোড ৯৯ প্রযোজ্য নয় এর জন্য ব্যবহার করুন এবং উত্তরের ক্ষেত্রে অন্যান্য হলে, দয়া করে উল্লেখ করুন।

Sl.	2b.2 H/O TB (যক্ষ্মার ইতিহাস)	2b.3 If Yes, How many days ago যদি হ্যাঁ হয়, কত দিন আগে	2b.4 Current TB status বর্তমানে যক্ষ্মার অবস্থা	2b.5 H/O Smoking (ধুমপানের ইতিহাস)	2b.6 Current Smoker (বর্তমানে কি ধুমপায়ী)	Substance use (নেশা জাতীয় দ্রব্য ব্যবহার)	Alcohol (মদ)	2b.9 Any Chronic illness (যেকোন দীর্ঘস্থায়ী অসুস্থতা)	Marital status (বৈবাহিক অবস্থা)	If female Pg. status যদি, মহিলা হয়, গর্ভের অবস্থা)	If Pg. Yes gestational (যদি গর্ভবতী হয়, তবে কত মাসের)	2b.10 Remarks (মন্তব্য)

Chronic illness: Diabetes, Heart disease, Kidney disease, Liver disease, Joint problem, Asthma, Cancer (দীর্ঘস্থায়ী অসুস্থতাঃ ডায়াবেটিস, হৃদ রোগ, কিডনির রোগ, যকৃত এর রোগ, জয়েন্টের সমস্যা, এ্যাজমা, ক্যান্সার)

Marital status: 1-Married, 2-Unmarried, 3-Widow, 4-Divorced (বৈবাহিক অবস্থাঃ ১-বিবাহিত, ২-অবিবাহিত, ৩-বিধবা, ৪-তালাকপ্রপ্ত)

3. Household background information (খানার তথ্য) [for the Ans. don't know use code-88 and for answer not applicable use 99, in case of ans. others: please specify]
এই উত্তরের জন্য কোড ৮৮ জানিনা এবং কোড ৯৯ প্রযোজ্য নয় এর জন্য ব্যবহার করুন এবং উত্তরের ক্ষেত্রে অন্যান্য হলে, দয়া করে উল্লেখ করুন।

Q no (প্রশ্ন নং)	Questions (প্রশ্ন)	Answer Options (উত্তরের বিকল্প)	Code (কোড)
3.1	What is the monthly income of the household? (খানার মাসিক আয় কত?)	In Taka (টাকায়)…………	If Don't know or refused to ans. skip to 3.5
3.2	What is the monthly expenditure of the household? (খানার মাসিক ব্যয় কত?)	In Taka (টাকায়)…………	

3.3	Number of rooms in the household (excluding kitchen)<br>পরিবারের কক্ষের সংখ্যা (রান্নাঘর বাদে)		
3.4	What kind of Toilet facility do your household members use?<br>(আপনার পরিবারের সদস্যরা কি ধরনের টয়লেট ব্যবহার করে?)	Flushed Toilet with septic tank…………………………1<br>সেপটিক ট্যাঙ্ক সহ ফ্লাশড টয়লেট<br>Toilet without septic tank……………………….....2<br>সেপটিক ট্যাঙ্ক ছাড়া টয়লেট<br>Improved Pit latrine……………………….3<br>উন্নত পিট ল্যাট্রিন<br>Open Pit latrine……………..4<br>খোলা পিট ল্যাট্রিন<br>Hanging toilet………………5<br>ঝুলন্ত ল্যাট্রিন<br>No toilet facility…………….6<br>টয়লেট এর সুবিধা নেই<br>Other…………………….....77<br>অন্যান্য	
3.5	Do you share the Toilet facility with any other households?<br>(আপনি কি অন্য কোন পরিবারের সাথে টয়লেট এর সুবিধা ভাগাভাগি করেন?)	1= Yes (হ্যাঁ), 0 =No (না)	
3.6	What is the main source of drinking water for members of<br>your household?<br>(আপনার পরিবারের সদস্যদের পানীয় জলের প্রধান উৎস কি?)	Piped water into dwelling……………………..1<br>বাসস্থানের মধ্যে পাইপের পানি<br>Piped water to Yard……………………...2<br>উঠানে পাইপ দিয়ে পানি<br>Public Tap………………….3<br>পাবলিক ট্যাপ<br>Tube well…………………...4<br>টিউবওয়েল<br>Dug well……………………5<br>কূপ<br>Springwate……………….....6<br>ঝর্ণার পানি<br>Rainwater……………..…...7<br>বৃষ্টির পানি<br>Surface water (pond/river/lake/canal)……..8<br>ভূ-পৃষ্ঠের পানি (পুকুর/নদী/লেক/খাল)<br>Other………………………77<br>অন্যান্য	
3.7	Do you do anything to the water to make it safer to drink? (পান করা পারি নিরাপদ করতে আপনি কি কিছু করেন?)	1= Yes (হ্যাঁ), 0 =No (না)	
3.8	Does your household have electricity? (আপনার বাড়িতে কি বিদ্যুৎ আছে?)	1= Yes (হ্যাঁ), 0 =No (না)	
3.9	Does your household have Solar Electricity (আপনার বাড়িতে কি সৌর বিদ্যুৎ আছে?)	1= Yes (হ্যাঁ), 0 =No (না)	

3.10	Does your household have the following things? (আপনার পরিবারের কি নিম্ন লিখিত জিনিস আছে?)		
3.10.1	A television? টেলিভিশন	1= Yes (হ্যাঁ), 0 =No (না)	
3.10.2	A Mobile phone? মোবাইল	1= Yes (হ্যাঁ), 0 =No (না)	
3.10.3	A Refrigerator? ফ্রিজ	1= Yes (হ্যাঁ), 0 =No (না)	
3.10.4	An Almira/wardrobe? আলমারি/ওয়াড্রপ	1= Yes (হ্যাঁ), 0 =No (না)	
3.10.5	A Fan? ফ্যান	1= Yes (হ্যাঁ), 0 =No (না)	
3.10.6	A computer/laptop? কম্পিউটার/ল্যাপটপ	1= Yes (হ্যাঁ), 0 =No (না)	
3.10.7	Other valuable goods?.. অন্যান্য মূল্যবান পণ্য	1= Yes (হ্যাঁ), 0 =No (না)	
3.11	What type of fuel does your household mainly use for Cooking? (আপনার পরিবার রান্নার জন্য প্রধানত কোন ধরনের জ্বালানী ব্যবহার করে?)	Electricity…………………...1 বিদ্যুৎ<br>LPG……………………2 এলপিজি<br>Natural Gas…………....3 প্রাকৃতিক গ্যাস<br>Biogas…………………4 বায়োগ্যাস<br>Kerosene……………….5 কেরোসিন<br>Charcoal………………..6 চরকোল<br>Wood…………………..7 কাঠ<br>Straw/Shrubs…………….8 খড়/ঝোপঝাড়<br>Animal dung……………..9 পশুর গোবর<br>Other…………………….77 অন্যান্য<br>No cooking………………0 রান্না করে না	**If ans. is 0, skip to 3.18**
3.12	Cooking Place? (রান্নার জায়গা?)	In the house……………….……..1 ঘরের মধ্যে<br>In a separate house……………………. 2 আলাদা ঘরে<br>Outdoors………………... 3 ঘরের বাইরে	
3.13	Does any member of this household own following things? (এই পরিবারের কোন সদস্য কি নিম্ন লিখিত জিনিসগুলির মালিক?)		
3.13.1	A car/microbus কার/মাইক্রোবাস	1= Yes (হ্যাঁ), 0 =No (না)	
3.13.2	An auto bike/CNG অটো রিক্সা/সিএনজি	1= Yes (হ্যাঁ), 0 =No (না)	
3.13.3	A motor cycle মটরসাইকেল	1= Yes (হ্যাঁ), 0 =No (না)	
3.13.4	A bicycle বাইসাইকেল	1= Yes (হ্যাঁ), 0 =No (না)	
3.13.5	A rickshaw/Van রিক্সা/ভ্যান	1= Yes (হ্যাঁ), 0 =No (না)	

3.14	Does this household own any poultry (এই পরিবারের কি কোন হাঁস-মুরগি আছে?)	1= Yes (হ্যাঁ), 0 =No (না)	
3.15	Does any member of this household have a bank account? (এই পরিবারের কোন সদস্যের কি ব্যাংক একাউন্ট আছে?)	1= Yes (হ্যাঁ), 0 =No (না)	
3.16	The main material of the floor (মেঝে তৈরির প্রধান উপাদান)		
3.17	The main material of the roof (ছাদ তৈরির প্রধান উপাদান)		
3.18	The main material of the exterior wall (বাহিরের দেয়ালের প্রধান উপাদান)		

4. Comments of the interviewer: প্রশ্নকর্তার মন্তব্যঃ

____

END
