# Supplementary material for: Active case finding for tuberculosis in tea gardens of Bangladesh: A cross-sectional survey
Source: PLoS One. 2025 Sep 29;20(9):e0333662. doi: 10.1371/journal.pone.0333662 (PMC12478949; doi:10.1371/journal.pone.0333662)
Supplement: S2 File — (PDF) [file pone.0333662.s002.pdf]

Active case finding (ACF) for Adult and Child
প্রাপ্তবয়স্ক এবং শিশুদের সক্রিয় রোগ অনুসন্ধান

1. Screening tool স্ক্রীনিং ফরম

1.1	Division (বিভাগ)	
1.2	District (জেলা)	
1.3	Upazilla (উপজেলা)	
1.4	Name of the Tea Garden (চা বাগানের নাম)	
1.5	Screening Date (স্ক্রিনিং এর তারিখ)	
1.6	Name of the household member (পরিবারের সদস্যের নাম)	
1.7	Age (বয়স) (Years / Month)	
1.8	Sex (লিঙ্গ) (Male=1, Female=2, 3rd gender=3)	
1.9	Father/Husband Name (পিতা/স্বামীর নাম)	
1.10	Contact No. (যোগাযোগের নম্বর)	
1.11	Sign/symptoms (চিহ্ন/লক্ষণ) (Adult)<br>1. Persistent Cough for ≥2 weeks (একনাগাড়ে ২ সপ্তাহ বা তার বেশি সময় ধরে কাশি)<br>2. Fever (জ্বর) (raising at evening/night)<br>3. Night sweats (রাতে ঘাম হওয়া)<br>4. Weight loss (ওজন কমে যাওয়া)<br>5. History of close contact with TB patient in last 12 months (গত ১২ মাস এর মধ্যে যক্ষ্মা রোগীর সংস্পর্শে আসার ইতিহাস)<br>6. Lump in neck, armpit or groin (ঘাড়, বগল বা কুঁচকিতে গুটি)	Sign and symptoms will appear according to age following the latest revised algorithms for both child and adult (TB screening tool) বয়স অনুযায়ী প্রাপ্তবয়স্ক এবং শিশুদের চিহ্ন ও উপসর্গ সর্বশেষ সংশোধিত এ্যাগরিদম অনুযায়ী প্রদর্শিত হবে (যক্ষ্মার অনুসন্ধান টুল)
	Sign/symptoms (Child) চিহ্ন/উপসর্গ (শিশু)<br>1. Persistent Cough for ≥2 weeks (একনাগাড়ে ২ সপ্তাহ বা তার বেশি সময় ধরে কাশি)<br>2. Fever for ≥2 weeks (২ সপ্তাহ বা তার বেশি সময় ধরে জ্বর)<br>3. Less playfulness (খেলাধুলা কমে যাওয়া)<br>4. Respiratory distress (শ্বাসকষ্ট)<br>5. Weight loss or not gaining weight (ওজন কমে যাওয়া বা ওজন না বাড়া)	

	7. History of close contact with TB patient in last 12 months (গত ১২ মাস এর মধ্যে যক্ষ্মা রোগীর সংস্পর্শে আসার ইতিহাস)<br>6. Lump in neck, armpit or groin (ঘাড়, বগল বা কুঁচকিতে গুটি)	
1.12	Presumptive for TB (অনুমিত যক্ষ্মা রোগী)	
1.13	Referred for TB test (যক্ষ্মা পরীক্ষার জন্য প্রেরণ)	

2. Investigation (পরীক্ষা)

2.1	X-ray (1=Y, 2=N) এক্স-রে (১=হ্যাঁ, ২=না)	If Y, then the image capture and observation option are given.
2.2	X-pert (1=Y, 2=N) এক্স-পার্ট (১=হ্যাঁ, ২=না)	If Y, then 'MTB detected' and 'MTB not detected'<br>If MTB detected the 'Rifampicin status' dropdown would show with options:<br>1. Rif Resistance not detected<br>2. Rif resistance detected<br>3. Rif resistance indeterminate
2.3	AFB-microscopy (1=Y, 2=N) এএফবি-মাইক্রোসকপি (১=হ্যাঁ, ২=না)	If Y, value: positive or negative
2.4	FNAC (1=Y, 2=N) এফএনএসি (১=হ্যাঁ, ২=না)	If Y, then the image capture and describe
2.5	Biopsy (1=Y, 2=N) বায়োপ্সি (১=হ্যাঁ, ২=না)	If Y, then the image capture and describe
2.6	MT ((1=Y, 2=N) এমটি (১=হ্যাঁ, ২=না)	If Y, the result dropdown value: positive or negative
2.7	Other (1=Y, 2=N) অন্যান্য (১=হ্যাঁ, ২=না)	If Y, then describe

3. Investigation result (পরীক্ষার ফলাফল)

3.1	Date (তারিখ)	Result Date. (পরক্ষার তারিখ)
3.2	TB detected (Y=1, N=2) যক্ষ্মা (১=হ্যাঁ, ২=না)	If Y, then
3.3	TB type (যক্ষ্মার ধরণ)	Either PTB or EPTB. If EPTB
3.4	Type (ধরণ)	Either B+ or CD case
3.5	Treatment Start (Y=1, N=2) চিকিৎসা শুরু (১=হ্যাঁ, ২=না)	If Y
3.6	TR Number (টিআর নম্বর)	Text box
3.7	Treatment Start date (চিকিৎসা শুরুর তারিখ)	Date field
